# West Nile virus infection in suspected febrile typhoid cases in Xinjiang, China

**DOI:** 10.1038/emi.2017.27

**Published:** 2017-06-07

**Authors:** Lei Cao, Shihong Fu, Zhi Lv, Chengjun Tang, Shiheng Cui, Xiaolong Li, Xiaoyan Gao, Minghua Li, Yuxi Cao, Wenwen Lei, Ying He, Huanyu Wang, Guodong Liang

**Affiliations:** 1 State Key Laboratory of Infectious Disease Prevention and Control, National Institute for Viral Disease Control and Prevention, Chinese Center for Disease Control and Prevention, Beijing 102206, China; 2 Collaborative Innovation Center for Diagnosis and Treatment of Infectious Diseases, Hangzhou 310058, China

**Dear Editor**,

West Nile virus (WNV) is a mosquito-borne virus.^1^ Infection with WNV generally causes a self-limiting disease, with central nervous system symptoms in severe cases. As the discovery of encephalitis caused by WNV in the United States in 1999, the virus has become a serious public health concern and a global emerging infectious disease.^2, 3^ However, most WNV-infected patients have only a transient fever with mild clinical manifestations, especially during the early stages of infection, which can easily be overlooked by clinicians and patients.^4^ Typhoid fever is an infectious intestinal disease caused by the bacterium *Salmonella typhi*. Severe cases may show numerous clinical manifestations, including rose-colored spots on the abdomen and hepatosplenomegaly. As with other acute infectious diseases, a high-grade fever may occur during the early stage of the disease; this can easily lead to misdiagnoses of these acute infectious diseases.^5, 6^

WNV was first isolated in mainland China from mosquito specimens in Jiashi County, Kashi Region, southern Xinjiang, following local outbreaks of viral meningitis and encephalitis caused by WNV.^7, 8^ Although WNV is an emerging pathogen in China and East Asia,^9^ there have been no recent reports of WNV encephalitis cases or epidemics in the region. Determining the incidence of WNV infection has become an important public health concern. During the investigation of WNV infections in the Kashi Region, Xinjiang, China, we found a high incidence of typhoid fever in the area, with large numbers of outpatients with fevers during the summer and autumn.^10^ Accurate clinical diagnosis is difficult without laboratory-specific detection because WNV infection and typhoid fever occur frequently during the summer and autumn, and both pathogens can cause a fever during the initial stages of infection. Therefore, in this study, serum was collected from suspected typhoid fever patients at Jiashi County Hospital, Xinjiang, China, during the acute phase of infection. The presence of WNV-specific immunoglobulin M (IgM) antibodies and neutralizing antibodies was examined, and tests were performed for typhoid fever. The results indicate that there were WNV-infected patients among those with typhoid fever.

Acute-phase serum was collected from 124 cases of suspected typhoid fever within 7 days at the Typhoid Fever Outpatient Department during the summer and autumn from July to September 2011, at Jiashi County Hospital, Xinjiang, China. Aliquots of serum were stored at –70 °C until use. The collected sera were examined for *Salmonella* infection and WNV infection simultaneously.^7^ *Salmonella* infection was detected by the Widal reaction (WR). Briefly, the agglutination test was performed against the H (flagellum) and O (cell) antigens of *Salmonella typhi* and ‘H’ antigens of *Salmonella paratyphi* A and *Salmonella paratyphi* B, as described previously.^11^ An acute serum sample was deemed positive when the antibody agglutination test reached O≥1:80, H≥1:160. WNV- and Japanese encephalitis virus (JEV)-specific IgM antibodies were detected by IgM-ELISAs (WNV IgM Capture DxSelect (Focus Diagnostics, Inc., Cypress, CA, USA) and JEV IgM Capture ELISA Kit (Panbio, Sinnamon Park, Queensland, Australia).^7^ For anti-WNV IgM antibody-positive patients, sera were collected during both the acute and recovery phases, in which neutralizing antibodies against WNV and JEV were detected by the 90% plaque-reduction neutralization test (PRNT_90_), respectively. Patients with a ≥4-fold difference in the WNV-neutralizing antibody titers of the convalescent serum/acute period serum samples were identified as positive for WNV infection.^12^

The results of the WR indicated that 81% (101/124) of the acute-phase serum samples were positive (that is, patients with typhoid fever), with WR serum geometric mean titers (95% confidence interval) of O 125 (110–142) and H 170.2 (160–186). At the same time, 34 samples were positive for anti-WNV IgM antibodies among 124 serum samples, suggesting the presence of WNV infection in patients with typhoid fever. To understand the levels of neutralizing antibodies against WNV in the acute and convalescent sera of WNV IgM-seropositive patients, we retrospectively investigated the WNV IgM-seropositive patients and collected the corresponding convalescent serum samples. In total, 21 convalescent serum samples were collected. In this study, paired acute-phase/convalescent serum samples from 21 patients were tested for WNV-neutralizing antibodies using the PRNT_90_. The results indicate that the WNV-neutralizing antibody titer during the acute phase of WNV infection was between 1:10 and 1:20, whereas that in the convalescent sera ranged from 1:40 to 1:320. From 21 paired serum samples, there were ≥4-fold differences in the WNV-neutralizing antibody titer between acute and convalescent serum samples from 11 patients. Those 11 patients who were positive for anti-WNV IgM antibodies and had a ≥4-fold difference in WNV-neutralizing antibody titer between the convalescent/acute serum samples were identified as being positive for WNV infection (Table 1). To eliminate cross-neutralization reactions between JEV and WNV, the same samples were examined for JEV-neutralizing antibodies at the same time. As we know, the existence of cross-reactions to one virus is usually affected by the antibody level against another virus. The results indicate that the JEV-neutralizing antibody results were negative (PRNT_90_<1:10), which were hypothesized due to low antibody levels against WNV, although some of the acute-phase serum samples were weakly positive for JEV-specific IgM antibodies. The detection of WNV nucleic acid by PCR and cell culture was performed using acute-phase serum samples from the 11 WNV-infected patients, all of which yielded negative results. The 11 WNV-infected patients were between the ages of eight and 88 years old. Among them, seven patients were ≥60 years old, three were 26–47 years old and one patient was eight years old. Elderly individuals were more susceptible, consistent with the observed age distribution of the WNV infection.^4, 13^ Overall, five of the 11 WNV patients were found to be seropositive by WR (clinical manifestations of fever, headache, sore throat, limb weakness and hepatosplenomegaly). Laboratory testing indicated that these five patients were co-infected with *Salmonella typhi* and WNV, whereas the remaining six patients were negative for *Salmonella typhi* and had only the WNV infection (Table 1).

Jiashi County (in the Kashi Region of western China) is an economically underdeveloped area. The natural environment is mainly desolate beach and desert with perennial drought; ponds have been built to collect rainwater and snowmelt for the irrigation of farmland and livelihood is suitable for mosquito-breeding areas and habitats for WNV-competent mosquitoes (for example, *Culex pipiens*). Typhoid is a common infectious disease in the area.^14^ To facilitate the timely treatment of typhoid patients, the Jiashi County Public Health Administrative Department set up a ‘Typhoid Fever Outpatient Service’ at the county hospital to provide specialized treatment for patients with typhoid fever. Patients with confirmed or suspected typhoid fever in the area during the summer were asked to first visit the Typhoid Fever Outpatient Service. The hospital conducts epidemiological investigations, provides medical examinations for patients, and collects acute-phase serum samples from suspected cases for laboratory testing. To prevent the spread and decrease the prevalence of typhoid fever, serum-positive patients are admitted to the hospital for treatment. Patients with a fever but a negative *Salmonella typhi* test have yet to be investigated. Patients with mild symptoms are suggested to undergo home observation, whereas severe cases are admitted to the hospital for further observation and treatment. The Typhoid Fever Outpatient Service of Jiashi County Hospital made it possible to identify patients with subclinical WNV infections in the present study.

In this study, 124 acute-phase serum samples were collected from patients with suspected typhoid fever at the Typhoid Fever Outpatient Service of Jiashi County Hospital. The laboratory test results indicated that 27% (34/124) were positive for anti-WNV IgM antibodies, of which 52% (11/21) showed a ≥4-fold difference in the WNV-neutralizing antibody titers of convalescent/acute serum samples, suggesting a higher incidence of WNV infection in patients with fever in Jiashi, Xinjiang, China.

Among the 11 patients with a WNV infection, 5 were positive for typhoid fever, indicating that they were co-infected with *Salmonella typhi* and WNV (Table 1). The clinical manifestations of *Salmonella typhi* and WNV co-infected patients were fever accompanied by a loss of appetite, nausea, vomiting, abdominal distension, abdominal pain and other digestive tract symptoms, whereas six patients with only a WNV infection exhibited either fever or fever with generalized symptoms. On the basis of early clinical manifestations of both groups, a WNV infection is accompanied by relatively simple clinical symptoms of fever and discomfort, whereas patients co-infected with *Salmonella typhi* and WNV show more complex symptoms, usually with gastrointestinal discomfort. This follow-up survey indicates that regardless of the patients’ infection status (*Salmonella typhi*, WNV or both), the patients were all alive two to three months after infection with no aggravation of their illness and no occurrence of viral encephalitis, suggesting that these patients, and especially those with WNV infection, showed only a transient fever and a mild clinical presentation.

This study revealed that WNV infection occurred sporadically in Jiashi County, with few cases clustered in the same village, suggesting a broad distribution for WNV in the area. Although the clinical symptoms of WNV infection were relatively mild and easily ignored, changes in the local climate (such as increased temperature and rainfall) would increase the density of local mosquitos (the vector), resulting in a greater prevalence and spread of WNV. Therefore, the disease spread and risk of epidemics would be increased. The detection and monitoring of WNV infection, especially in cases of unexplained fever, are important to accurately assess the burden of WNV infection and to prevent epidemics.

## Figures and Tables

**Table 1:** West Nile virus infection in suspected febrile typhoid cases, Xinjiang, China

**Gender**	**Age (years)**	**Illness onset**	**Sampling time interval (days)**	**Symptoms/diagnosis**^a^	**Widal reaction**	**JEV** **RT-PCR**	**JEV IgM-ELISA**	**JEV PRNT**_**90**_	**WNV** **RT-PCR**	**WNV IgM-ELISA**	**WNV PRNT**_**90**_
Male	60	20110728	1	Fever/unknown fever^b^	−	−	+	<1:10	−	+	<1:10
			85				−	<1:10		−	1:40
Female	27	20110730	1	Fever and pneumonia/unknown fever	−	−	−	<1:10	−	+	1:20
			83				−	<1:10		+	1:80
Male	67	20110731	1	Fever/unknown fever	−	−	−	<1:10	−	+	1:10
			84				−	<1:10		+	1:80
Male	63	20110810	1	Fever, poor appetite, nausea, vomiting, pharyngeal hyperemia, abdominal distension and abdominal pain/typhoid fever	H1:160;O1:320	−	+	<1:10	−	+	1:40
			73				−	<1:10		−	1:160
Female	62	20110812	1	The body temperature rises slowly, acute bronchitis/typhoid fever	H1:320;O1:320	−	+	<1:10	−	+	1:40
			71				−	<1:10		+	1:320
Male	72	20110824	1	Prolonged high fever, muscle and joint pain, weak, pharyngeal hyperemia, abdomen distended and painful/typhoid fever	H1:320;O1:640	−	−	<1:10	−	+	1:20
			62				−	<1:10		+	1:320
Female	46	20110824	1	Fever, poor appetite, abdominal distension and abdominal pain, diarrhea, rose spots appeared one week after onset/typhoid fever	H1:640;O1:640	−	+	<1:10	−	+	1:20
			60				−	<1:10		+	1:160
Male	66	20110824	1	Fever, malaise, headache, insomnia/unknown fever	−	−	+	<1:10	−	+	1:40
			60				+	<1:10		+	1:320
Male	26	20110824	1	Fever and pneumonia/unknown fever	−	−	+	<1:10	−	+	1:20
			61				−	<1:10		+	1:80
Female	88	20110828	1	Fever, headache, poor appetite, lumbago, muscle and joint pain, and limb weakness/typhoid fever	H1:320;O1:320	−	+	<1:10	−	+	1:20
			64				−	<1:10		+	1:160
Female	8	20110828	1	Fever/unknown fever	−	−	+	<1:10	−	+	1:20
			56				−	<1:10		−	1:80

Abbreviations: enzyme-linked immunosorbent assay, ELISA; immunoglobulin M, IgM; Japanese Encephalitis virus, JEV; plaque-reduction neutralization test, PRNT_90_; reverse transcription PCR, RT-PCR; West Nile virus, WNV.

a Symptoms/diagnosis: symptoms: patient complained/diagnosis: clinical diagnosis without pathogenic detection (typhoid and West Nile Virus).

b Unknown fever: means unknown fever (typhoid and West Nile Virus).
